# Analysis of Germline Variants in *CDH1*, *IGFBP3*, *MMP1*, *MMP3*, *STK15* and *VEGF* in Familial and Sporadic Renal Cell Carcinoma

**DOI:** 10.1371/journal.pone.0006037

**Published:** 2009-06-24

**Authors:** Christopher Ricketts, Maurice P. Zeegers, Jan Lubinski, Eamonn R. Maher

**Affiliations:** 1 Cancer Research UK Renal Molecular Oncology Group, Department of Medical and Molecular Genetics, University of Birmingham School of Medicine, Edgbaston, Birmingham, United Kingdom; 2 Unit of Genetic Epidemiology, Department of Public Health and Epidemiology, University of Birmingham, Birmingham, United Kingdom; 3 Department of Genetics and Pathology, International Hereditary Cancer Center, Pomeranian Medical University, Szczecin, Poland; 4 West Midlands Regional Genetics Service, Birmingham Women's Hospital, Edgbaston, Birmingham, United Kingdom; 5 Department of Complex Genetics, Cluster of Genetics and Cell Biology and Nutrition and Toxicology Research Institute Maastricht (NUTRIM), Maastricht University, Maastricht, the Netherlands; University of Montreal, Canada

## Abstract

**Background:**

The investigation of rare familial forms of kidney cancer has provided important insights into the biology of sporadic renal cell carcinoma (RCC). In particular, the identification of the von Hippel Lindau (VHL) familial cancer syndrome gene (*VHL*) provided the basis for the discovery that *VHL* is somatically inactivated in most sporadic clear cell RCC. Many cases of familial RCC do not have mutations in known RCC susceptibility genes and there is evidence that genetic modifiers may influence the risk of RCC in VHL disease patients. Hence we hypothesised that low-penetrance functional genetic variants in pathways related to the VHL protein (pVHL) function might (a) modify the phenotypic expression of VHL disease and/or (b) predispose to sporadic RCC.

**Methodology/Principal Findings:**

We tested this hypothesis for functional polymorphisms in *CDH1* (rs16260), *IGFBP3* (rs2854744), *MMP1* (rs1799750), *MMP3* (rs679620), *STK15* (rs2273535) and *VEGF* (rs1570360). We observed that variants of *MMP1* and *MMP3* were significant modifiers of RCC risk (and risks of retinal angioma and cerebellar haemangioblastoma) in VHL disease patients. In addition, higher frequencies of the *MMP1* rs1799750 2G allele (p = 0.017, OR 1.49, 95%CI 1.06–2.08) and the *MMP1*/*MMP3* rs1799750/rs679620 2G/G haplotype (OR 1.45, 95%CI 1.01–2.10) were detected in sporadic RCC patients than in controls (n = 295).

**Conclusions/Significance:**

These findings (a) represent the first example of genetic modifiers of RCC risk in VHL disease, (b) replicate a previous report of an association between *MMP1*/*MMP3* variants and sporadic RCC and (c) further implicate *MMP1*/*MMP3*-related pathways in the pathogenesis of familial and sporadic RCC.

## Introduction

Familial renal cell carcinoma (RCC) accounts for 2–3% of all patients with RCC, but the investigation of rare familial forms of kidney cancer has provided important insights into the pathogenesis of non-familial RCC. Thus germline mutations in the von Hippel-Lindau (VHL) disease tumour suppressor gene (*VHL*) are the most common cause of familial renal cell carcinoma and somatic inactivation of *VHL* occurs in most sporadic clear cell RCC [1]–[4]. Hence the *VHL* tumour suppressor gene product (pVHL) has a key “gatekeeper” role in the pathogenesis of RCC [5]. Inherited mutations in a variety of other genes including *MET*, *FLCN, FH* and *SDHB* may also be associated with inherited RCC [6]–[7]. However, many cases of familial RCC do not have a mutation in known RCC susceptibility genes [8]–[9]. Furthermore, genetic modifier effects may influence the risk of RCC in VHL disease [10]–[11]. These observations suggest that unknown genetic factors contribute to the development of RCC. A variety of approaches have been employed in order to identify novel genetic causes of RCC including the mapping and characterisation of RCC-associated constitutional translocations (see 12 and references within) and genetic association studies [13]-[15]. We hypothesised that functional genetic variants in pathways related to pVHL function might modify the phenotypic expression of VHL disease and/or predispose to sporadic RCC. We tested this hypothesis for polymorphisms in *CDH1*, *IGFBP3*, *MMP1*, *MMP3*, *STK15* and *VEGF*. Polymorphic variants in *CDH1* (rs16260 at c.-160), *IGFBP3* (rs2854744 at c.-202), *MMP1* (rs1799750 at c.-1607) and *VEGF* (rs1570360 at c.-1154) had previously been reported to alter promoter function [16]-[19]. In addition, missense substitutions in *STK15* and *MMP3* (rs2273535 (p.Phe31Ile) and rs679620 (p.K45E) respectively) were also analysed.

## Methods

### Patient Groups

219 patients and unaffected carriers with germline VHL mutations from 134 kindreds were analysed for the “VHL modifier analysis” study. We also analysed a cohort of 317 Polish sporadic RCC patients and 295 Polish normal controls that were matched to the patients by sex and approximate year of birth and were ascertained from the same region. The Polish sporadic RCC patients consisted of 226 men (mean age at diagnosis of RCC 60.7 years (range 26–89 years)) and 117 women (mean age at diagnosis of RCC 60.2 years (range 17–84 years)). The Polish normal controls consisted of 204 men (mean age 64 years (range 40–90 years)) and 117 (mean age 63.6 years (range 24–91 years)). All controls had a negative cancer family history. The study protocol was approved by the local Research Ethics bodies and participants gave informed consent.

### Molecular Genetic Studies

*VHL* mutation analysis in the VHL patient cohort was performed by direct sequencing and MLPA analysis and the mutation analysis results have been reported previously [20]. Genotyping of candidate functional polymorphisms in *CDH1* (rs16260 promoter variant c.-C160A), *IGFBP3* (rs2854744 promoter variant c.-C202A), *MMP1* (rs1799750 promoter variant c.-1607 2G/1G), *MMP3* (rs679620 c.A198G → Lys45Glu), *STK15* (rs2273535 c.T91A → Phe31Ile) and *VEGF* (rs1570360 promoter variant c.-G1154). were performed by competitive allele specific PCR system (KASPar, KBiosciences). Details of primers and reaction conditions are available on request.

### Statistical analysis

For the “VHL modifier analysis” study Kaplan-Meir survival curves were constructed and Cox regression analysis was performed to determine with the effect of different *CDH1*, *IGFBP3*, *MMP1*, *MMP3*, *STK15* and *VEGF* alleles on age at onset of renal, retinal and cerebellar tumours in VHL patients.

For analysis of sporadic RCC patients and controls, deviation from Hardy Weinberg proportions for the genotypes of both markers and linkage disequilibrium between marker alleles was tested by χ2 tests. We calculated odds ratios (OR) and corresponding 95% confidence intervals (95%CI) using logistic regression for genotypic, allelic and haplotypic analyses. We estimated the false-positive report probability (FRFP) for statistically significant observations using the methodology described by Wacholder et al [21]. This method assumes that the prior probability that the association between a genetic variant and a disease is real is likely to be influenced by knowledge of the biological function of a gene and previous evidence that an association exists. The FRFP is calculated for a range of prior probabilities (50% to 0.1%). A prior probability of 50% might be appropriate when there is strong biological plausibility and consistent previous evidence for an association, whilst a prior probability of 0.1% would be appropriate when there is no biological or previous supporting evidence for an association. We selected the prior probabilities for the calculation of FRFP for our data according to the pre-existing evidence for likely association with RCC. Thus the *MMP1* promoter polymorphism (rs1799750) has been shown previously to influence expression of the *MMP1* gene [22] and has been reported previously to be associated with risk of RCC and other cancers in [23]–[26]. In the light of this prior probabilities of 25% (and 10%) were assigned [21]. The *STK15* rs2273535 missense substitution has been reported previously to alter the STK15 function [27] and to be associated with numerous number of different cancer types [28]–[31]. However, in a previous study, no statistically significant association between rs2273535 and RCC risk was detected [13]. Hence more conservative prior probabilities (1%–10%) were selected for the calculation of FRFP. In accordance with Wacholder et al [21] a standard FRFP cut-off of less than 0.5 was selected with a cut-off of 0.2 being considered more stringent.

A Bayesian statistical method for reconstructing uncertain haplotypes was applied by using the program PHASE [32] version 2.1. For allelic and haplotypic analyses, the Huber sandwich estimator of variance [33] have been calculated to take into account the clustering of two chromosomes within individuals. The most frequent haplotype (1G/G) was used as the reference haplotype. An omnibus likelihood ratio based test was performed to investigate whether the regression coefficient of at least one of the haplotypes in the model is not equal to zero. In the results the omnibus test showed no significance (p = 0.09). All statistical analyses were performed using Stata 9.0 [34].

## Results

### VHL Modifier Analysis and variants in *CDH1*, *IGFBP3*, *MMP1*, *MMP3*, *STK15* and *VEGF*

**Figure 1:**
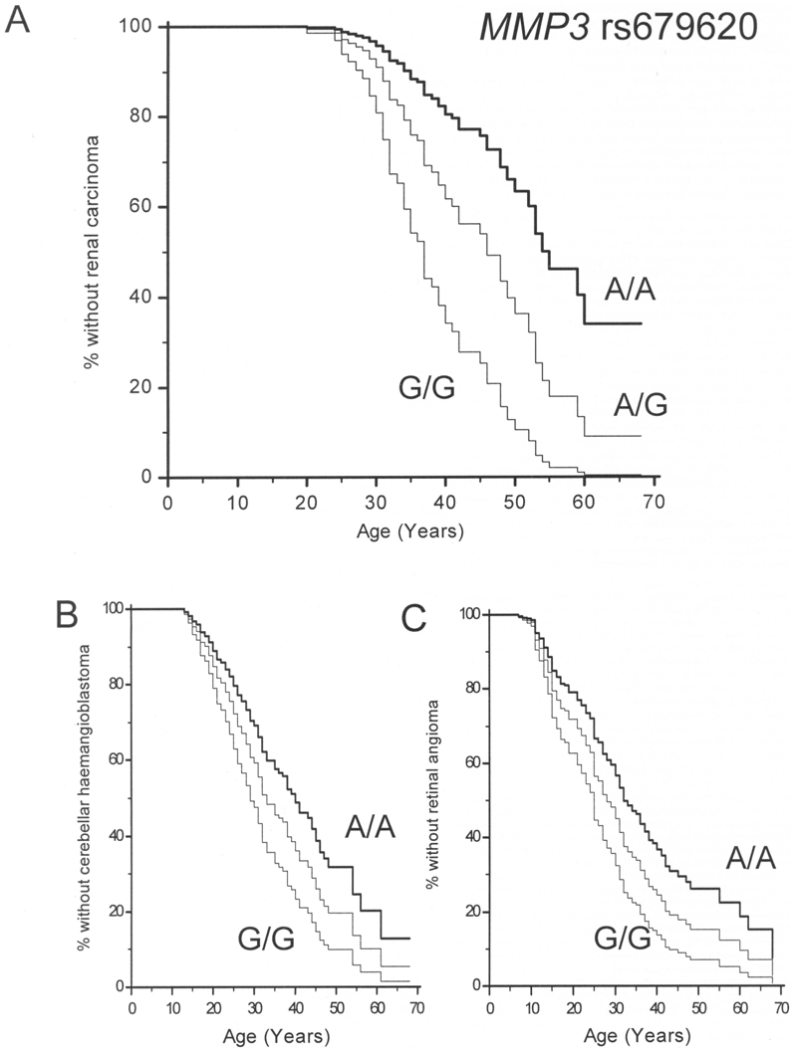
Effect of MMP3 rs679620 genotype on tumour risk in VHL disease. Panel A: VHL patients homozygous for the p.45Glu MMP3 allele (rs679620 c.198G) had an earlier onset of RCC than those homozygous for the p.45Lys MMP3 allele (rs679620 c.198A) and heterozygotes had an intermediate risk. Similar associations were also found for onset of cerebellar haemangioblastoma and retinal angioma (see panels B and C respectively).

**Figure 2:**
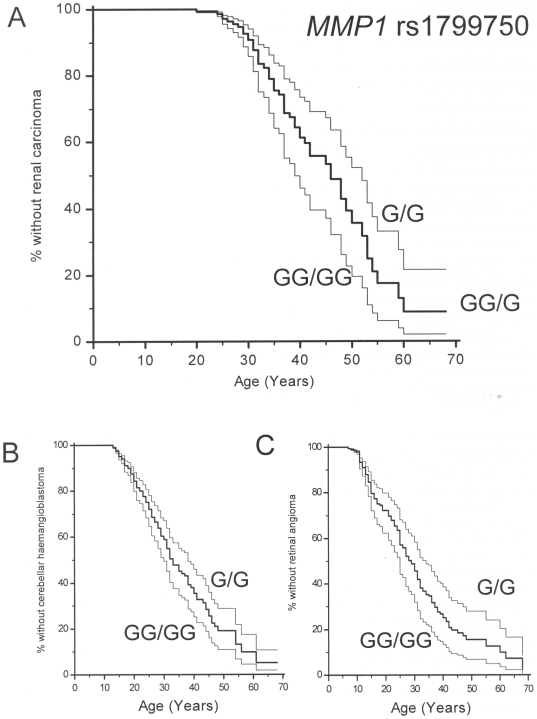
Effect of MMP1 rs1799750 genotype on tumour risk in VHL disease. Panel A: Homozygotes for the “high-risk” MMP1 allele (rs1799750, c.-1607 2G) had an earlier onset of RCC than patients homozygous for the “low risk” MMP1 allele (rs1799750, c.-1607 1G), with heterozygotes having an intermediate risk. Similar associations were also found for onset of cerebellar haemangioblastoma and retinal angioma (see panels B and C respectively).

Analysis of genotypes of candidate functional modifiers in 6 genes was performed using a Cox proportional hazard model analysis such that all the variables were entered into the regression model and then non-significant (P>0.1) variables removed. For RCC risk, the final model (overall χ2 = 11.9336 P = 0.0026) retained only *MMP1* and *MMP3* (P = 0.0291 and 0.0006731) and excluded SNPs in *CDH1*, *IGFBP3*, *STK15* and *VEGF* as significant variables. VHL patients homozygous for the p.45Glu *MMP3* allele (rs679620 c.198G) had an earlier onset of RCC than those homozygous for the p.45Lys *MMP3* allele (rs679620 c.198A) and heterozygotes had an intermediate risk (see Figure 1A). Similarly, homozygotes for the “high-risk” *MMP1* allele (rs1799750, c.-1607 2G) had an earlier onset of RCC than patients homozygous for the “low risk” *MMP1* allele (rs1799750, c.-1607 1G), with heterozygotes having an intermediate risk (see Figure 2A).

To determine if the observed modifier effects of *MMP1* and *MMP3* variants extended to other features of VHL disease, we repeated the Cox proportional hazard model analysis for age at diagnosis of retinal and cerebellar haemangioblastomas. This gave similar results, albeit less significant, to those for RCC. Thus for retinal angiomas the final model (overall χ2 = 7.04 P = 0.0296) retained *MMP1* (rs1799750) and *MMP3* (rs679620) (P = 0.012 and P = 0.041 respectively) and likewise, for cerebellar haemangioblastoma (overall model fit χ2 = 5.99 P = 0.0499) only *MMP1* (rs1799750) and *MMP3* (rs679620) were retained (P = 0.028 and P = 0.055 respectively) and *CDH1* (rs16260), *IGFBP3* (rs2854744), *STK15* (rs2273535) and *VEGF* (rs1570360) were excluded. As for RCC, homozygotes for the “high-risk” *MMP1* allele (rs1799750, c.-1607 2G) had an earlier age at diagnosis of retinal angioma and cerebellar haemangioblastoma than patients homozygous for the “low risk” *MMP1* allele (rs1799750, c.-1607 1G) (Fig 2B and 2C) and homozygotes for the p.45Glu *MMP3* allele (rs679620 c.198G) had earlier age at diagnosis of retinal angioma and cerebellar haemangioblastoma than those homozygous for the p.45Lys *MMP3* allele (rs679620 c.198A (see Fig 1B and Fig 1C).

### RCC Association Study for variants in *CDH1*, *IGFBP3*, *MMP1*, *MMP3*, *STK15* and *VEGF*

317 RCC patients and 295 controls were analysed for six polymorphic variants in six candidate RCC susceptibility genes. All genotypes were in Hardy Weinberg equilibrium. Comparison of allele/genotype frequencies demonstrated significant differences between patients and controls for *MMP1* rs1799750 and *STK15* rs2273535 polymorphic variants (see Table 1).

**Table 1:** Genotyping data for sporadic RCC cases.

Gene	SNP	Controls		Sporadic RCCs		Fisher's P-value	OR (95%CI)
**CDH1**	**rs16260**	**C**	**A**	**C**	**A**	0.537	1.08 (0.85–1.38)
		444 (72%)	174 (18%)	458 (70%)	194 (30%)		
		**CC/CA**	**AA**	**CC/CA**	**AA**	0.777	1.11 (0.63–1.94)
		284 (92%)	25 (8%)	297 (91%)	29 (9%)		
		**CC**	**CA/AA**	**CC**	**CA/AA**	0.579	1.10 (0.81–1.50)
		160 (52%)	149 (48%)	161 (49%)	165 (51%)		
**IGFBP3**	**rs284744**	**C**	**A**	**C**	**A**	0.288	1.13 (0.90–1.43)
		376 (64%)	214 (36%)	381 (61%)	246 (39%)		
		**CC/CA**	**AA**	**CC/CA**	**AA**	0.125	1.45 (0.90–2.33)
		262 (89%)	33 (11%)	268 (85%)	49 (15%)		
		**CC**	**CA/AA**	**CC**	**CA/AA**	0.452	1.14 (0.82–1.58)
		114 (39%)	181 (61%)	113 (36%)	204 (64%)		
**MMP1**	**rs1799750**	**1G**	**2G**	**1G**	**2G**	**0.049**	**1.25 (1.00–1.56)**
		306 (49%)	322 (51%)	279 (43%)	367 (57%)		
		**2G1G/1G1G**	**2G2G**	**2G1G/1G1G**	**2G2G**	**0.030**	**1.47 (1.04–2.07)**
		234 (75%)	80 (25%)	215 (67%)	108 (33%)		
		**1G1G**	**2G2G/2G1G**	**1G1G**	**2G2G/2G1G**	0.194	1.20 (0.82–1.76)
		72 (23%)	242 (77%)	64 (20%)	259 (80%)		
**MMP3**	**rs679620**	**G**	**A**	**G**	**A**	0.866	1.03 (0.82–1.28)
		330 (53%)	288 (47%)	342 (53%)	306 (47%)		
		**GG/GA**	**AA**	**GG/GA**	**AA**	0.286	1.09 (0.63–1.94)
		243 (78%)	66 (22%)	250 (77%)	74 (23%)		
		**GG**	**GA/AA**	**GG**	**GA/AA**	0.509	0.99 (0.70–1.40)
		87 (28%)	222 (72%)	92 (28%)	232 (72%)		
**STK15**	**rs2273535**	**A**	**T**	**A**	**T**	0.063	0.78 (0.60–101)
		464 (74.5%)	158 (25.5%)	519 (80%)	137 (20%)		
		**AA/AT**	**TT**	**AA/AT**	**TT**	0.725	0.82 (0.24–2.79)
		293 (94%)	18 (6%)	312 (95%)	16 (5%)		
		**AA**	**AT/TT**	**AA**	**AT/TT**	**0.044**	**0.71 (0.52–0.98)**
		171 (55%)	140 (45%)	207 (63%)	121 (37%)		
**VEGF**	**rs1570360**	**G**	**A**	**G**	**A**	0.159	1.18 (0.94–1.49)
		422 (67%)	206 (33%)	411 (63%)	237 (37%)		
		**GG/GA**	**AA**	**GG/GA**	**AA**	0.415	1.23 (0.78–1.95)
		276 (92%)	38 (92%)	277 (92%)	47 (92%)		
		**GG**	**GA/AA**	**GG**	**GA/AA**	0.202	1.23 (0.90–1.68)
		146 (92%)	168 (92%)	134 (92%)	190 (92%)		

For the *MMP1* rs1799750 polymorphic variant, the “high-risk” *MMP1* allele (c.-1607 2G, that was associated with an earlier onset of RCC in VHL patients) was significantly increased in the Polish sporadic RCC patients compared to controls (p = 0.049 OR 1.25, 95%CI 1.003–1.55)(See Table 1). Additionally the homozygous genotype containing the “high-risk” *MMP1* allele (rs1799750, c.-1607 2G) was significantly increased in the Polish sporadic RCC patients compared to controls (p = 0.030 OR 1.47, 95%CI 1.04–2.07)(See Table 1). According to the criteria of Wacholder et al [21], this result remained robust given the appropriate prior probabilities of 25% (FRFP = 0.139) and 10% (FRFP = 0.362).

Although there were no significant differences between RCC patients and controls for *MMP3* rs679620, there was strong evidence of linkage disequilibrium between *MMP1* rs1799750 and *MMP3* rs679620 (D' = 0.50, χ^2^ = 92.4, P<0.001). In the light of this haplotype analysis was undertaken. Comparison of haplotype risks to the reference haplotype rs1799750, c.-1607 1G/rs679620, 198G demonstrated a significant increased risk for rs1799750, c.-1607 2G/rs679620, 198G (OR 1.45, 95%CI 1.01–2.10) but not for rs1799750, c.-1607 1G/rs679620, 198A (OR 0.9, 95%CI 0.62–1.31) or rs1799750, c.-1607 2G/rs679620, 198A (OR 1.21, 95%CI 0.95–1.55) haplotypes. Thus the combination of the two alleles associated with increased tumour risk in VHL patients was also associated with the highest risk.

For the *STK15* rs2273535 polymorphic variant, the homozygous genotype containing the p.31Ile *STK15* allele (c.91A) was significantly increased in the Polish sporadic RCC patients compared to controls (p = 0.044 OR 1.40, 95%CI 1.02–1.92) (See Table 1). The addition of the Polish familial RCC patients increased the significance (p = 0.027 OR 1.42, 95%CI 1.04–1.94). This result remained robust at a prior probability of 10% (FRFP = 0.365) but not with a prior probability of 1% (FRFP = 0.865).

## Discussion

Our findings suggest that functional SNPs in *MMP1*/*MMP3* can influence susceptibility to RCC in familial (VHL disease) and sporadic patients and that *MMP1* rs1799750 and *MMP3* rs679620 genotypes can also influence the risk of retinal angioma and cerebellar haemangioblastoma in VHL disease. The ability to invade normal tissue and metastasise is a key feature of malignant neoplasms and the matrix metalloproteinase family of zinc-dependent enzymes (MMPs) have a key role in degrading the extracellular matrix and facilitating tissue invasion by cancer cells. In addition, MMPs may regulate availability of growth factors and enhance angiogenesis [35]–[37]. Matrix metalloproteinase 1 (MMP1) has a specific ability to degrade type-I collagen (the most abundant substrate in the tumour surrounding stroma) and has been implicated in tumour invasion and metastasis [38], whereas MMP3 degrades a broader range of substrates (e.g. fibronectin, laminin, collagens III, IV, IX, and X, and cartilage proteoglycans), and may also affect the expression of other MMPs [39]. The rs1799750 *MMP1* 1G/2G promoter polymorphism has been shown to influence *MMP1* transcription in both normal fibroblasts and in melanoma cells [22]. An inverse correlation between MMP1 expression and cancer prognosis has been reported in many cancers [40]–[42] and the rs1799750 variant has been linked with an increased risk of developing lung, ovarian, colorectal, and head and neck cancers [24]–[26]. In addition, Hirata et al (2003) reported an increased frequency of the rs1799750 2G variant in RCC cases (n = 119) from Japan compared to population controls (n = 210) [23]. We have replicated this finding in a larger cohort of RCC patients from a different ethnic group.

The rs679620 A *MMP3* p.K45E polymorphism in the matrix metalloproteinase 3 (stromelysin-I) gene has been associated previously with differences in MMP3 activity and has been linked to cancer susceptibility in some studies [43]–[45]. Association studies of *MMP1* and *MMP3* SNPs are complicated by the colocation of these two genes within a *MMP* gene cluster at 11q22.3 and we detected evidence of strong linkage disequilibrium between rs1799750 and rs679620. A previous study demonstrated an association between a rs1799750 and rs679620 haplotype consisting of the *MMP1* 1G/2G polymorphism and the *MMP3* Glu45Lys polymorphism. Hence we investigated both variants separately and as a combined haplotype. This demonstrated that the strongest link with RCC was associated with the *MMP1* rs1799750 2G/*MMP3* rs679620 G haplotype. Again this is consistent with the findings of Hirata et al (2004) who reported that the frequency of the same allelic haplotype was significantly higher in the RCC patients of Japanese descent than in the controls (crude OR = 1.95, 95%CI = 1.31–2.91).

We did not find any evidence that the tested genetic variants in *CDH1* (rs16260), *IGFBP3* (rs2854744), *VEGF* (rs1570360) and *STK15* (rs2273535) influenced risk of RCC in VHL disease patients. Unlike *MMP1* and *MMP3*, none of these variants has previously been associated with RCC risk in sporadic patients [13]. Thus there would appear to be a good correlation between variants that can modify RCC risk in VHL and those that have been reported to be associated with RCC risk in sporadic RCC patients. However, there are two caveats to this observation. Firstly it is possible that that these variants might be demonstrated an effect in larger studies. Secondly, we detected a statistically significant association between a genetic variant (rs2273535) in *STK15* and RCC in sporadic patients. STK15 (Aurora-A) is a serine/threonine kinase essential for chromosome segregation and cytokinesis. Overexpression of STK15 is common in many cancers and is associated with centrosome amplification, chromosome instability and cell transformation [46]. Previously the *STK15* (rs2273535, p.Phe31Ile) variant was found to alter the potency of STK15 transformation [27] and was reported to be associated with cancer risk in a number of cancer types including ovarian, colorectal, breast, oesophagus and lung [28]–[31]. Although, Hammerschmeid et al [13] did not detect a significant association between the p.Phe31Ile variant and RCC, the patient and control study groups (n = 156 and n = 158 respectively) were less than half as many as those analysed by us and so further analysis of a larger groups is indicated.

We estimated the false-positive reporting probability (FPRP) for our results by incorporating a range of prior probabilities that specific polymorphisms are associated with RCC risk [21], [47]. The association between the MMP1 genotype and RCC risk was extremely robust with a prior probability of 25% (FRFP = 0.139) (and remained robust if the prior probability was reduced to 10%). Given that the pre-existing evidence for an association between the STK15 p.Phe31Ile variant and RCC was less secure a lower prior probability (10%) was selected. Whilst the FRFP was still in favour of a real association (FRFP = 0.365) at this prior probability, it should be emphasised that the putative *STK15* association should be confirmed in larger cohorts.

Previously, we reported that the phenotypic expression of VHL disease is influenced by modifier effects and that patients with more severe retinal angiomatosis also had increased age-related risks of cerebellar haemangioblastomas and RCC [10]. Subsequently, we reported that a functional variant in the VHL target gene *CCND1* influenced risk of retinal angiomas and central nervous system hemangioblastomas (but not RCC) in VHL disease patients [11]. Hence the finding of an association between *MMP1* and *MMP3* genotypes and RCC risk represents the first examples of genetic modifiers of RCC risk in VHL disease. In addition, the observation that *MMP1* and *MMP3* genotypes also appeared to influence retinal angioma and cerebellar haemangioblastoma risks is consistent with our previous report suggesting shared genetic modifiers of retinal angiomatosis, cerebellar haemangioblastomas and RCC risks in VHL disease [10]. Previously, pVHL was reported to downregulate metalloproteinases, such as MMP1, and upregulate MMP inhibitors (TIMPs) [48], so it is not unreasonable to suggset that genetic variants in MMP/TIMP pathways might influence tumourigenesis in VHL disease. In addition, as somatic VHL inactivation occurs in most clear cell RCC, and this histopathology accounts for ∼75% of all sporadic RCC, it is not unexpected that genetic modifiers of VHL disease RCC risk, might also function as low penetrance RCC susceptibility alleles (e.g. the association between *MMP1/MMP3* hapolotypes and RCC). Both STK15 and pVHL have been linked to p53 function [49]–[50], but although we found evidence for an association between *STK15* rs2273535 31Ile genotype and RCC susceptibility, there was no apparent evidence of modifier effects in VHL disease. Further studies are required to confirm the role of *STK15* variation in RCC susceptibility but it could be that the influence of germline STK15 variants is predominantly on a VHL-independent pathway of renal tumourigenesis. Our findings suggest that functional genetic variants in VHL-related pathways should be further evaluated as candidate genetic modifiers in VHL disease and as RCC susceptibility alleles. The identification of common variants associated with RCC risk in VHL and sporadic patients could provide further insights into RCC biology and highlight candidate familial RCC genes suitable for resequencing studies to detect rare high penetrance mutations.
